# *In vitro* assessment of immunomodulatory and anti-*Campylobacter* activities of probiotic lactobacilli

**DOI:** 10.1038/s41598-019-54494-3

**Published:** 2019-11-29

**Authors:** Khaled Taha-Abdelaziz, Jake Astill, Raveendra R. Kulkarni, Leah R. Read, Afsaneh Najarian, Jeffrey M. Farber, Shayan Sharif

**Affiliations:** 1 Department of Pathobiology, Ontario Veterinary College, University of Guelph, Guelph, ON N1G 2W1 Canada; 2 Pathology Department, Faculty of Veterinary Medicine, Beni-Suef University, Al Shamlah, 62511 Beni-Suef Egypt; 3 Department of Population Health and Pathobiology, College of Veterinary Medicine, North Carolina State University, Raleigh, North Carolina 27519 US; 4 Canadian Research Institute for Food Safety (CRIFS), Guelph, ON, N1G 2W1, ON N1G 2W1 Canada

**Keywords:** Bacterial infection, Antimicrobials

## Abstract

The present study was undertaken to assess the antimicrobial activity of *Lactobacillus* spp. (*L. salivarius*, *L. johnsonii*, *L. reuteri*, *L. crispatus*, and *L. gasseri*) against *Campylobacter jejuni* as well as their immunomodulatory capabilities. The results demonstrated that lactobacilli exhibit differential antagonistic effects against *C. jejuni* and vary in their ability to elicit innate responses in chicken macrophages. All lactobacilli exerted inhibitory effects on *C. jejuni* growth, abrogated the production of the quorum sensing molecule autoinducer-2 (AI-2) by *C. jejuni* and inhibited the invasion of *C. jejuni* in human intestinal epithelial cells. Additionally, all lactobacilli, except *L. reuteri*, significantly reduced the expression of virulence-related genes in *C. jejuni*, including genes responsible for motility (*flaA, flaB*, and *flhA*), invasion (*ciaB*), and AI-2 production (*luxS*). All lactobacilli enhanced *C. jejuni* phagocytosis by macrophages and increased the expression of interferon (IFN)-γ, interleukin (IL)-1β, IL-12p40, IL-10, and chemokine (CXCLi2) in macrophages. Furthermore, *L. salivarius, L. reuteri, L. crispatus*, and a mixture of all lactobacilli significantly increased expression of the co-stimulatory molecules CD40, CD80, and CD86 in macrophages. In conclusion, these findings demonstrate that lactobacilli possess anti-*Campylobacter* and immunomodulatory activities. Further studies are needed to assess their protective efficacy against intestinal colonization by *C. jejuni* in broiler chickens.

## Introduction

In recent years, there has been an increasing interest in the use of antimicrobial alternatives to reduce the burden of foodborne pathogens in poultry. To date, several antimicrobial alternatives have been identified, including prebiotics, probiotics and their by-products, herbs and their extract, enzymes, organic acids, essential oils, bacteriophages, antimicrobial peptides, nanoparticles and other immune-stimulants such as Toll-like receptor (TLR) ligands^1–4^. Among these, probiotics have gained increasing attention due to their actions not only in reducing enteric pathogen burden but also their ability to modulate immune responses and thereby, enhancing gut health in poultry.

Probiotics are a group of microorganisms, often referred to as beneficial bacteria that when added to poultry feed or water confer various health benefits^4,5^. In addition to their role in improving poultry growth performance^6^ and gut health^7^, numerous studies have demonstrated that probiotic supplementation, with either single or multiple species, can prevent or reduce intestinal colonization by foodborne pathogens in chickens, such as *Salmonella*^8,9^ and *Campylobacter*^10^. Growing evidence indicates that probiotic bacteria exert their antimicrobial effects through various mechanisms, including competitive exclusion of potentially pathogenic microorganisms by competing for nutrients and mucosal adhesion sites, production of antimicrobial metabolites such as volatile fatty acids and bacteriocins^11,12^, and modulation of the immune system^13–17^ and microbiota composition^18,19^. In the context of bacterial infections, previous studies have shown that the addition of *Lactobacillus salivarius* to chicken feed or drinking water can prevent *Salmonella enterica* serovar Enteritidis colonization^8,9^. In another study, oral administration of a mixture of probiotics (*Lactobacillus acidophilus*, *Bifidobacterium bifidum*, and *Streptococcus faecalis*) reduced cecal colonization by *Salmonella enterica* serovar Typhimurium in chickens^15^.

Despite their ability to reduce *Salmonella* colonization, the use of probiotics to control enteric *C. jejuni* colonization has shown variable success. For example, a 6 log_10_ reduction in *C. jejuni* colonization was observed in broiler chickens that received multispecies probiotics, consisting of *Enterococcus*, *Pediococcus*, *Lactobacillus*, and *Bifidobacterium*^10^. In contrast, another study reported that a probiotic mixture of *L. acidophilus*, *Bacillus subtilis*, and *Enterococcus faecium* could not significantly reduce cecal colonization of *C. jejuni* in broiler chickens^20^. The variability observed in these studies may be due to large variations in the antimicrobial and/or immune-stimulatory activities of the different probiotics. For instance, a recent study demonstrated that of 117 isolates of *Bacillus* and *Lactobacillus* spp., only 26 exhibited inhibitory activity against *C. jejuni in vitro*^21^. Furthermore, a number of studies have demonstrated differential abilities of *Lactobacillus* spp. (*L. acidophilus*, *L. reuteri* and *L. salivarius*) to enhance phagocytosis and modulate immune responses of chicken macrophage^16,17,22^.

Thus, prior to evaluating the protective efficacy of probiotics against *C. jejuni* colonization in chickens, a rigorous investigation of the anti-*C. jejuni* potentials of probiotic candidates, as well as characterization of their immunomodulatory properties are warranted. Therefore, the present study was undertaken to assess the anti-*C. jejuni* activities of five *Lactobacillus* spp. (*L. salivarius, L. reuteri, L. crispatus, L. johnsonii, L. gasseri*) of chicken origin and their immunomodulatory capabilities.

## Results

### Lactobacilli inhibited the growth of *C. jejuni*

**Figure 1:**
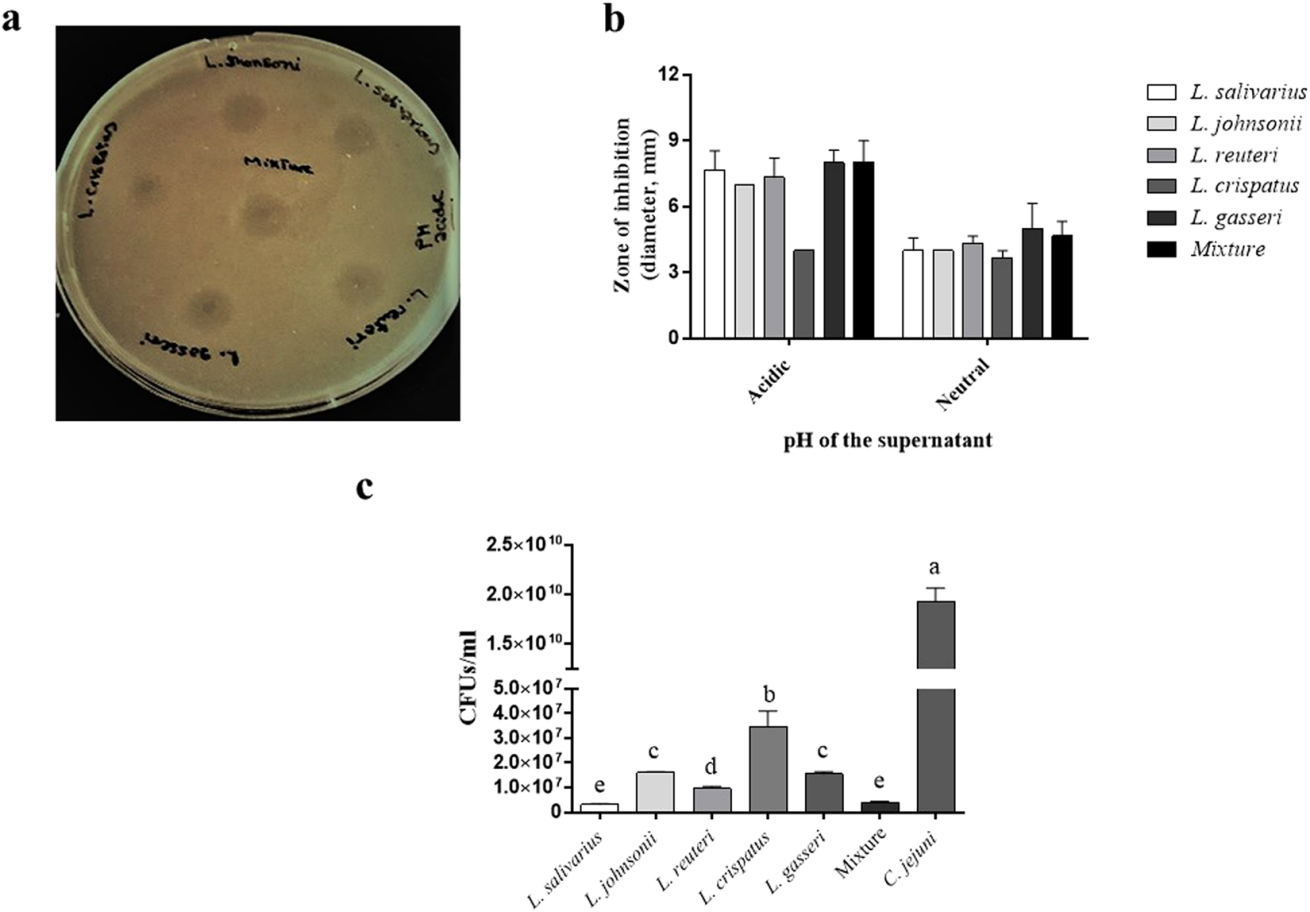
Inhibitory and bactericidal activity of both the live culture and cell-free supernatants of lactobacilli against *C. jejuni*. (**a**) Agar gel diffusion assay: 10^8^ CFUs *C. jejuni* were mixed with 30 mL of MH agar and poured into a round Petri dish. Approximately 3 mm diameter holes were punched in the agar and 20 μL of the cell-free supernatant of each *Lactobacillus* spp., or a mixture of all lactobacilli was added to the holes. Plates were overlaid with 10 mL of MH agar and incubated at 41 °C under microaerophilic conditions. After an incubation period of 40–48 h, the dimeters of the zones of inhibition (a clear ring around the well) were measured. (**b**) Comparison between the diameter of the inhibition zones induced by natural-acidic and neutralized lactobacilli supernatants. This assay was conducted in triplicate with similar results. (**c**) Killing assay: equal volumes of 10^7^ CFUs of each *Lactobacillus* spp. or the mixture of all species in MRS and 10^7^ CFUs of *C. jejuni* in MH broth were co-incubated overnight under microaerophilic conditions. Untreated *C. jejuni* culture was used as a positive control. Subsequently, 100 µL of each culture was serially diluted and streaked onto MH agar. Plates were incubated at 41 °C under microaerophilic conditions and CFUs of *C. jejuni* were enumerated after 40–48 h of incubation. Graphical data are presented as mean ± standard error of the mean (SEM). Bars (within a time point) which are marked by the same letter did not differ significantly (Duncan’s multiple range tests, P > 0.05). This assay was conducted in triplicate and repeated twice.

An agar radial diffusion assay was used to evaluate the inhibitory activity of the cell-free supernatants of the five *Lactobacillus* spp., either as a single species or in combination, against *C. jejuni*. The results revealed that both the naturally-acidic and neutralized cell-free supernatants of all *Lactobacillus* spp. were capable of inhibiting *C. jejuni* growth; however, varying levels of *C. jejuni* inhibition were observed. The inhibition zones induced by the naturally-acidic supernatants of all *Lactobacillus* spp. were not significantly different from their respective neutralized supernatants. There was no significant increase in the size of the inhibition zone when the supernatants were combined (Fig. 1a,b).

The killing assay was used to quantitatively measure the bactericidal activity of both the live culture and cell-free supernatants of lactobacilli against *C. jejuni*. The results showed that both the naturally-acidic and the neutralized supernatants of each *Lactobacillus* sp. or their combination completely inhibited the growth of *C. jejuni* (data not shown). With respect to the live cultures, each *Lactobacillus* sp. or their combination significantly reduced the number of *C. jejuni* compared to the untreated culture of *C. jejuni*. Treatment effects with either *L. salivarius* or the lactobacilli mixture was found to be superior as determined by a significantly higher reduction in *C. jejuni* numbers compared to the other treatment groups (Fig. 1c).

### Lactobacilli down-regulated the expression of virulence-related genes in *C. jejuni*

To identify the suitable internal reference genes, the expression patterns and PCR amplification efficiencies of 6 housekeeping genes of *C. jejuni* were screened using LightCycler® 480 Software. The results revealed constant expression levels of *ilvC, rpoA, thuC*, and *rrs* following exposure to a single or a mixture of lactobacilli, compared to the other housekeeping genes. In regard to *gyrA* and *sylD*, there were low to undetectable levels of expression and, therefore, these two genes were excluded from subsequent analysis. The efficiency of *ilvC, rpoA, thiC* and *rrs* was 0.85, 0.89, 0.92, and 0.92, respectively. Further analysis was conducted using SASqPCR to assess and rank the expression stability of these genes revealing that *thiC* and *rrs* were the most stable genes, whereas *ilvC* and *rpoA* were the least stable genes (Table 1). Based on these findings, *rrs* was selected as a housekeeping gene to normalize the expression of target gene*s*.

**Table 1:** PCR amplification efficiency and stability of *C. jejuni* reference genes.

Gene	Slope			Intercept			RSQ	E	Rank	
	Value	StdErr	P value	Value	StdErr	P value				
ilvc	−3.7365	0.0755	3.08E-11	37.64	0.1849	3.80E-16	0.996	0.851	1	
rpoA	−3.6185	0.0499	1.47E-12	37.641	0.1224	1.40E-17	0.998	0.889	2	
rrs	−3.521	0.0115	1.46E-17	30.066	0.0282	6.72E-22	0.999	0.923	3	
thic	−3.5155	0.0862	1.45E-10	35.182	0.2113	1.89E-15	0.995	0.925	4	

To gain insight into the role of the lactobacilli in the attenuation of *C. jejuni* virulence, the expression of several virulence genes of *C. jejuni* (*flaA, flaB, flhA, flhB, cadF, ciaB, iamA, cdtA*, and *luxS*) were measured following exposure to either a single or a mixture of *Lactobacillus* spp. In general, lactobacilli varied in their ability to attenuate the expression of these genes.

**Figure 2:**
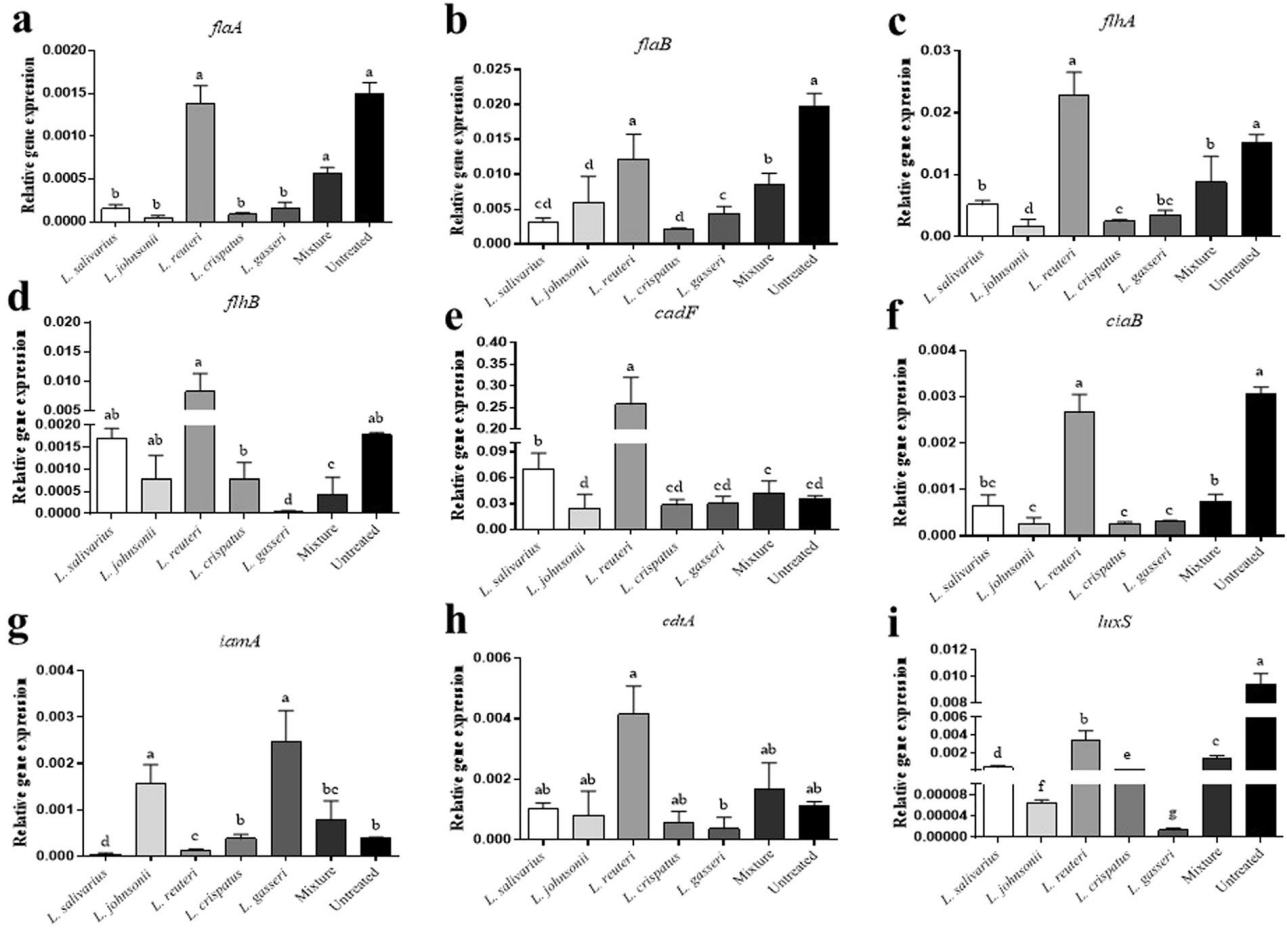
Relative gene expression of *C. jejuni* virulence-related genes. Equal volumes of 10^7^ CFUs of *C. jejuni* in MH broth and 10^8^ CFUs of each *Lactobacillus* sp., or the mixture of all species, or MRS medium were incubated at 41 °C for 24 h under microaerophilic conditions. Quantitative real time-PCR was used to measure the relative expression of genes responsible for motility such as *flaA* (**a**), *flab* (**b**), *flhA* (**c**), and *flhB* (**d**), adhesion such as *cadF* (**e**), invasion such as *ciaB* (**f**) and *iamA* (**g**), cytotoxin production such as *cdtA* (**h**) and autoinducer production such as luxS (**i**). Expression levels of all genes were calculated relative to the reference gene *rrs* (16S RNA ribosomal subunit) using 2^−ΔΔCT^ method. Graphical data are presented as mean ± standard error of the mean (SEM). Bars (within a time point) which are marked by the same letter did not differ significantly (Duncan’s multiple range tests, P > 0.05). This assay was conducted in triplicate.

Expression of *flaA* and *flaB* was downregulated at 24 h following exposure to all *Lactobacillus* spp., except *L. reuteri*, as compared to the unexposed *C. jejuni* group. No significant changes were observed in *flaA* and *flaB* expression between *L. reuteri*-exposed *C. jejuni* and unexposed *C. jejuni* group (Fig. 2a,b). Treatment with the mixture of lactobacilli significantly reduced the expression of *flaB* but not *flaA*. Expression of levels of *flhA* were significantly decreased following exposure to the lactobacilli mixture and all single *Lactobacillus* spp. except for *L. reuteri* (Fig. 2c). For *flhB*, expression levels were significantly reduced following exposure to *L. gasseri* and the lactobacilli mixture, whereas no significant differences were observed between the rest of *Lactobacillus* spp.-exposed groups and the unexposed *C. jejuni* group (Fig. 2d).

Expression of *cadF* was significantly enhanced following exposure to *L. salivarius and L. reuteri*, whereas no significant changes were observed after exposure to *L. johnsonii, L. gasseri, L. crispatus*, and the mixture of lactobacilli (Fig. 2e).

Expression of *ciaB* was significantly decreased following exposure to the lactobacilli mixture and all single *Lactobacillus* spp., except *L. reuteri*, compared to the unexposed *C. jejuni* group (Fig. 2f). *iamA* expression levels were significantly elevated following exposure to *L. johnsonii* and *L. gasseri*, whereas exposure to *L. salivarius and L. reuteri* significantly reduced the expression of *iamA* as compared to the unexposed *C. jejuni* group. Exposure to *L. crispatus* and the lactobacilli mixture, however, did not alter *cadf* expression compared to the unexposed *C. jejuni* group (Fig. 2g).

Expression of *cdtA* was significantly elevated following exposure to *L. reuteri*, whereas no significant changes were observed following exposure to the rest of *Lactobacillus* spp. and the lactobacilli mixture compared to the unexposed *C. jejuni* group (Fig. 2h).

Expression of *luxS* was significantly reduced following exposure to all *Lactobacillus* spp., either singly or as a mixture, compared to the unexposed *C. jejuni* group. Additionally, *L. gasseri* resulted in the highest reduction of *luxS* expression of all treatment groups (Fig. 2i).

### Lactobacilli inhibited the quorum sensing signals of *C. jejuni* by suppressing the production of AI-2

**Figure 3:**
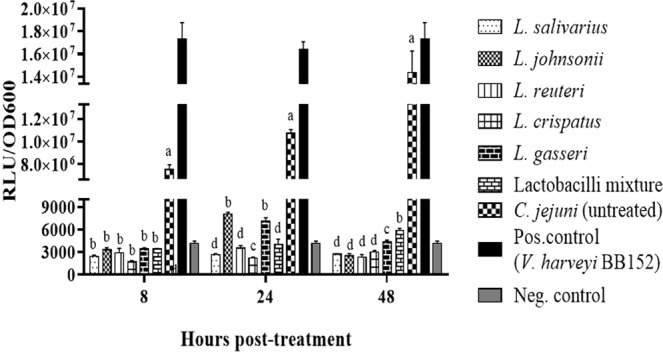
Levels of extracellular AI-2 produced by *C. jejuni* following co-incubation with lactobacilli at 41 °C for 24 h under microaerophilic conditions. *V. harveyi* bioluminescence assay was used to measure luminescence production. In a 96-well black clear bottom plate, 90 µL of the diluted *V. harveyi* BB170 and 10 µL of the filtered cell-free culture supernatant of both the treated and non-treated culture of *C. jejuni* were added to the wells. The supernatant from *V. harveyi* BB152 was used as a positive control and AB medium was used as a negative control. Maximal bioluminescence was observed at 13 hours after incubation with the lactobacilli supernatants. Data are presented as mean ± standard error of the mean (SEM) of the relative light unit (RLU) per unit of absorbance (cell density; OD 600 nm). Bars (within a time point) which are marked by the same letter did not differ significantly (Wilcoxon signed-rank test, P > 0.05). This assay was conducted in triplicate and repeated twice with similar results.

Quantitative measurement of the quorum sensing molecule AI-2 was performed by monitoring *V. harveyi* luminescence following treatment with the culture supernatants of *C*. *jejuni*, done in the presence or absence of lactobacilli for various time points. The results showed a consistent increase in AI-2 production across the time course in the untreated *C. jejuni* group, whereas treatment with the supernatants of *Lactobacillus* spp., either singly or as a mixture, resulted in abrogation of AI-2 production to levels comparable to those of the negative control group. At 8 h post-incubation, there were no significant differences in AI-2 production between treatment groups, whereas different effects between the treatment supernatants were observed at 24 and 48 h post-incubation (Fig. 3).

### Lactobacilli reduced *C. jejuni* adherence to and invasion of human intestinal epithelial cells

**Figure 4:**
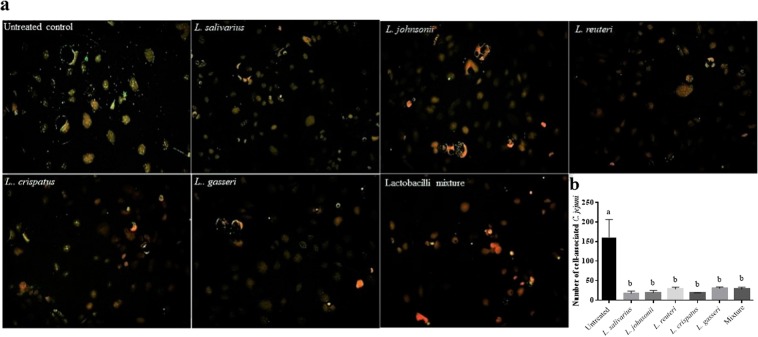
Lactobacilli reduced *C. jejuni* adherence to or invasion of human intestinal epithelial cells (Caco-2 cells). Caco-2 cells were seeded in 6-well plates at density 4 × 10^5^ cells/well in EMEM and incubated at 37 °C in a humidified 5% CO_2_ environment until reaching 90% confluency followed by 10^7^ CFUs/mL of FITC-labelled *C. jejuni* were added alone or simultaneously with 10^7^ CFUs/mL of each *Lactobacillus* sp. or a mixture of all species to cultured cells and incubated for 2, 5 and 8 h. ImageJ software was used to count the number of *C. jejuni* associated with Caco-2 cells. The green color represents the *C. jejuni*, while the red color represents the nucleus. This assay was conducted in triplicate and repeated twice with similar results.

The capacity of *Lactobacillus* spp. to inhibit the invasion of *C. jejuni* in Caco-2 cells was investigated by incubating the cells with fluorescein isothiocyanate (FITC)-labelled *C. jejuni* alone or in combination with lactobacilli and visualizing the invaded fluorescent *C. jejuni*. The results indicated that co-incubation of each *Lactobacillus* sp. or their combination with *C. jejuni* resulted in a significant reduction of *C. jejuni* adherence to and invasion of Caco-2 cells as demonstrated by fewer number of *C. jejuni* associated with cells co-cultured with *C. jejuni* and lactobacilli, compared to cells co-cultured with *C. jejuni* alone (Fig. 4a,b).

### Lactobacilli enhanced nitric oxide (NO) production in chicken macrophages

**Figure 5:**
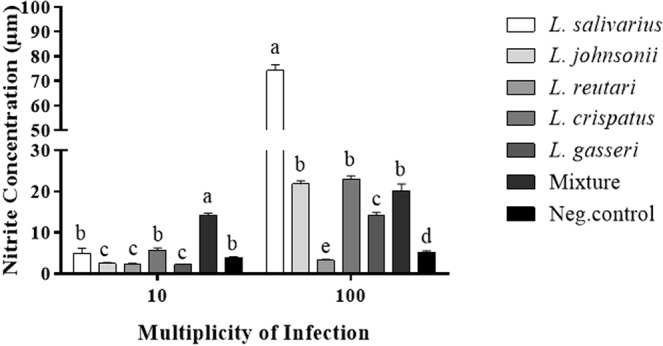
Lactobacilli enhanced the production of nitric oxide by macrophages. MQ-NCSU cells were seeded in 24-well plates at density 4 × 10^5^ cells/well in LM-HAHN medium and incubated at 40 °C in a humidified 5% CO_2_ environment. Following incubation for 3 h, cells were stimulated with 10 or 100 MOI of a single or a mixture of heat-killed *Lactobacillus* spp. in DMEM and incubated at 41 °C in a humidified 5% CO_2_ environment for 24 h. Supernatants were collected, and NO production was measured by Griess assay. Graphical data are presented as mean ± standard error of the mean (SEM). Bars (within a time point) which are marked by the same letter did not differ significantly (Wilcoxon signed-rank test, P > 0.05). This assay was carried out using five replicates.

The immunomodulatory activity of lactobacilli on chicken macrophages was evaluated by measuring NO production, which is an indicator of macrophage activation, following exposure to either a single or a mixture of heat-killed lactobacilli. Treatment of macrophages with a single heat-killed *Lactobacillus* sp., at a dose of 10 MOI, did not significantly increase levels of the NO relative to the untreated cells. The combination of *Lactobacillus* spp., however, synergistically enhanced the production of NO, as compared to the untreated cells. Treatment of macrophages with 100 MOI of a single species of all heat-killed lactobacilli, except *L. reuteri*, significantly enhanced NO production by macrophages compared to the untreated cells. The treatment of macrophages with *L. salivarius* induced a significantly higher NO production as compared to the other single species of *Lactobacillus* and their combination, whereas treatment with *L. reuteri* significantly reduced NO production compared to the untreated control. There were no statistically significant differences in the NO levels between the lactobacilli mixture and *L. johnsonii* or *L. crispatus* alone (Fig. 5).

### Lactobacilli enhanced the phagocytic activity of chicken macrophage-like cells

**Figure 6:**
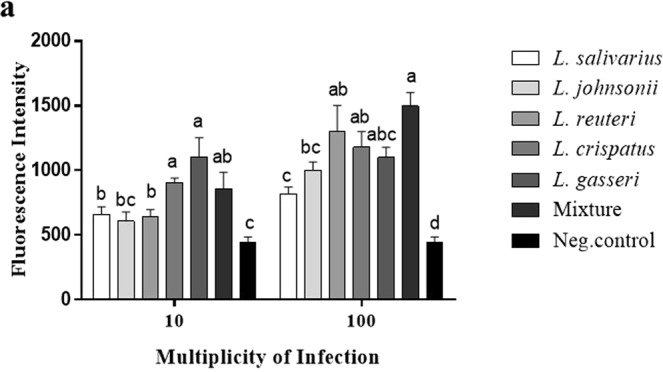
Lactobacilli enhanced macrophage phagocytosis of IgG-FITC coated latex beads. MQ-NCSU cells were seeded, in 6 replicates, into 96-well black clear bottom polystyrene plates at density 1 × 10^5^ cells/well in LM-HAHN medium and incubated for 3 h. Cells were then stimulated with 10 or 100 MOI of a single or a mixture of heat-killed *Lactobacillus* spp. in DMEM along with the Latex BeadsRabbit IgG-FITC complex and incubated at 41 °C in a humidified 5% CO_2_ environment. The fluorescence intensity was read in a fluorescence plate reader at an excitation of 485 nm and an emission of 535 nm. Graphical data are presented as mean ± standard error of the mean (SEM). Bars (within a time point) which are marked by the same letter did not differ significantly (Duncan’s multiple range tests, P > 0.05). This assay was carried out using six replicates.

**Figure 7:**
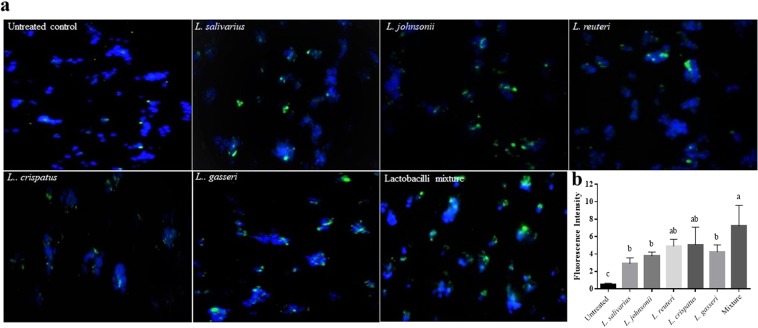
Lactobacilli enhanced macrophage phagocytosis of FITC-labelled *C. jejuni*. MQ-NCSU cells were seeded into 6-well plates containing glass coverslips at density 8 × 10^5^ cells/well in LM-HAHN medium as described above. After treatment with 100 MOI of heat-killed lactobacilli, cells were incubated at 41 °C in a humidified 5% CO_2_ environment for 2 h. Subsequently, cells were infected with 8 × 10^7^ CFUs of *C. jejuni*/well in DMEM medium. The invasion of fluorescent *C. jejuni* was visualized by fluorescence microscopy (**b**). The fluorescence intensity of the phagocytosed *C. jejuni* was measured by imageJ (**c**). This assay was conducted in triplicate and repeated twice with similar results. Graphical data are presented as mean ± standard error of the mean (SEM). Bars (within a time point) which are marked by the same letter did not differ significantly (Duncan’s multiple range tests, P > 0.05).

The effects of lactobacilli on the phagocytic activity of macrophages were evaluated using latex beads and FITC-labelled *C. jejuni*. Fluorescent latex beads were used to determine the optimal MOI of lactobacilli required for the enhancement of macrophage phagocytosis. The results indicated that 100 MOI of each *Lactobacillus* sp. or their mixture, significantly enhanced the phagocytic capacity of macrophages compared to 10 MOI (Fig. 6). Therefore, MOI of 100 was chosen as an optimal dose to enhance *C. jejuni* uptake by macrophages. Pre-treatment of macrophages with 100 MOI heat-killed *Lactobacillus*, either singly or as a mixture, significantly enhanced phagocytosis of FITC-labelled *C. jejuni* by macrophages as demonstrated by the high number of *C. jejuni* associated with cells that were pre-treated with lactobacilli compared to the untreated cells (Fig. 7a,b). Macrophages treated with the mixture of lactobacilli exhibited a higher phagocytic activity than those treated with *L. salivarius* and *L. johnsonii*. and *L. gasseri*, whereas no significant differences were observed between the other treatments and the mixture of lactobacilli.

### Lactobacilli modulated cytokine gene expression in chicken macrophages

**Figure 8:**
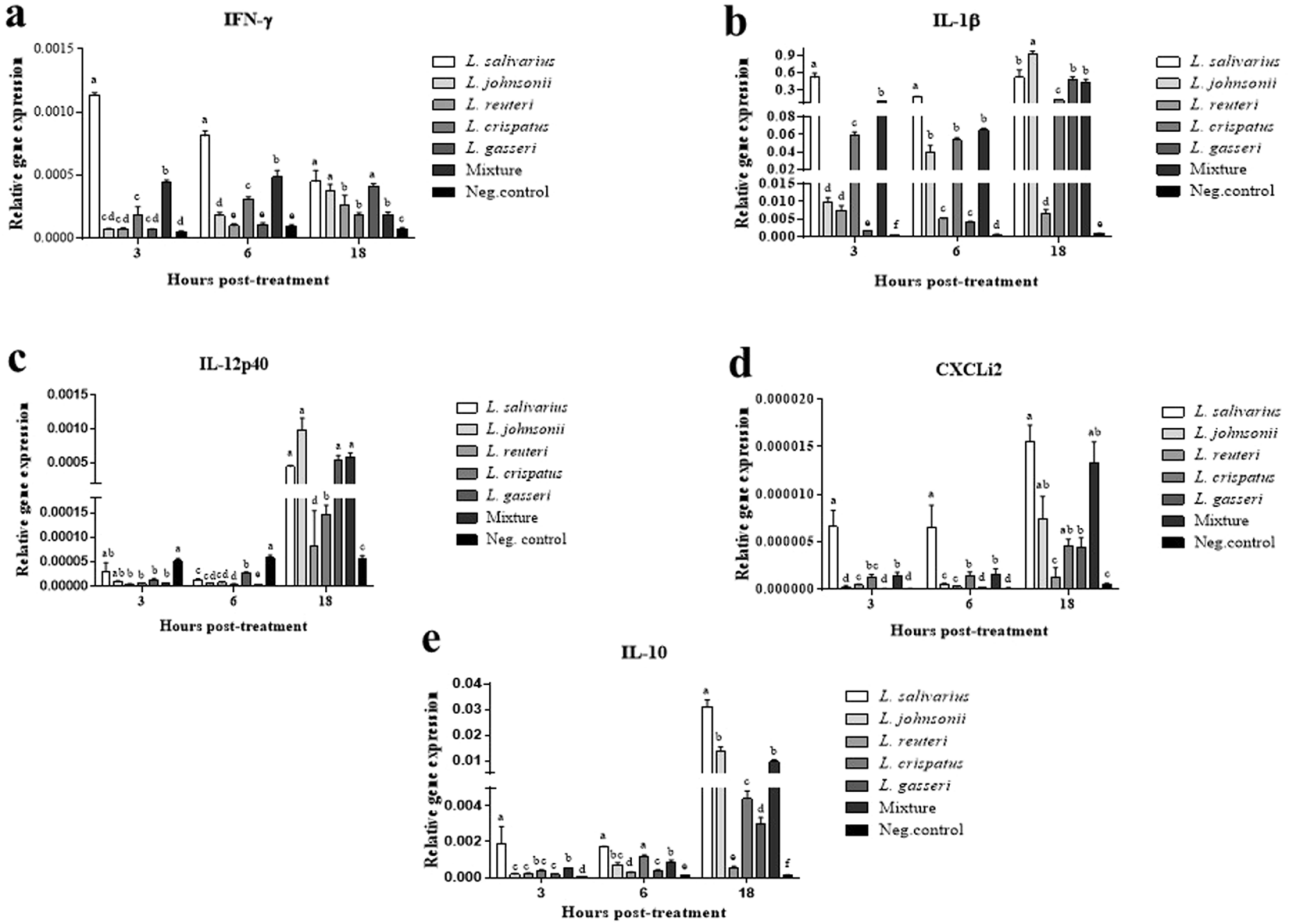
Relative gene expression of IFN-γ (**a**), IL-1β (**b**), IL-12p40 (**c**), CXCLi2 (**d**), and IL-10, (**e**) in macrophages at 3, 6, and 18 h following exposure to heat-killed lactobacilli. Expression levels of all target genes were calculated relative to the housekeeping gene β-actin using 2^−ΔΔCT^ method. Graphical data are presented as mean ± standard error of the mean (SEM). Bars (within a time point) which are marked by the same letter did not differ significantly (Duncan’s multiple range tests, P > 0.05).

We explored the kinetics of cytokine and chemokine gene expression in chicken macrophages following exposure to lactobacilli. Irrespective of the time point, treatment with either a single or a mixture of lactobacilli significantly enhanced the expression of IFN-γ (Fig. 8a), pro-inflammatory cytokines (IL-1β, IL-12p40, Fig. 8b,c), a pro-inflammatory chemokine (CXCLi2, Fig. 8d), and a regulatory cytokine (IL-10, Fig. 8e). Importantly, treatment with *L. salivarius*, *L. johnsonii*, and the mixture of lactobacilli consistently induced substantially higher levels of expression of these cytokines and chemokine followed by *L. gasseri* and *L. crispatus*, compared to the untreated control group. The expression of all cytokines and chemokines in the group treated with *L. reuteri* were statistically higher than the untreated control group, but significantly lower than the other treatment groups.

### Lactobacilli enhanced the expression of costimulatory and antigen presentation molecules of macrophages

**Figure 9:**
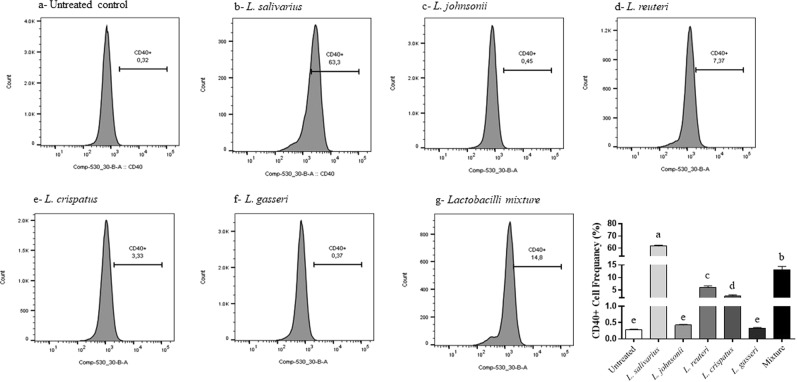
Expression of macrophage costimulatory molecule CD40 following treatment with heat-killed lactobacilli. MQ-NCSU cells were seeded, in triplicates, into 24-well plates and then treated with 100 MOI of heat-killed lactobacilli. After 24 hours of incubation, cells were harvested and stained with unlabelled anti-CD40 followed by labelled anti-mouse IgG1 secondary antibodies. Data analysis was performed using FlowJo software. Graphical data are presented as mean ± standard error of the mean (SEM). Bars (within a time point) which are marked by the same letter did not differ significantly (Duncan’s multiple range tests, P > 0.05).

**Figure 10:**
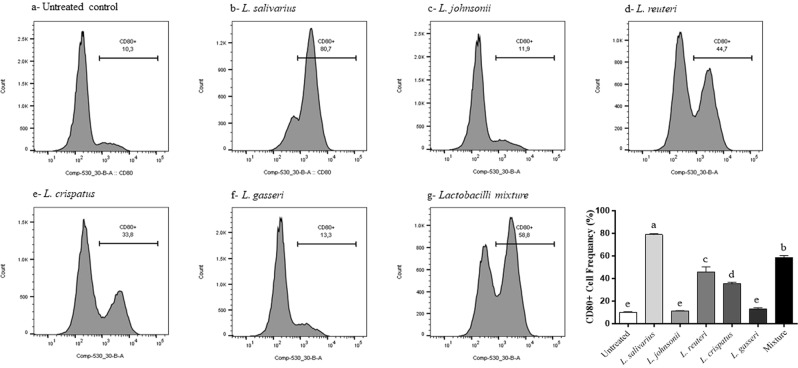
Expression of macrophage costimulatory molecule CD80 following treatment with heat-killed lactobacilli. MQ-NCSU cells were seeded, in triplicates, into 24-well plates and then treated with 100 MOI of heat-killed lactobacilli. After 24 hours of incubation, cells were harvested and stained with unlabelled anti-CD80 followed by labelled anti-mouse IgG1 secondary antibodies. Data analysis was performed using FlowJo software. Graphical data are presented as mean ± standard error of the mean (SEM). Bars (within a time point) which are marked by the same letter did not differ significantly (Duncan’s multiple range tests, P > 0.05).

**Figure 11:**
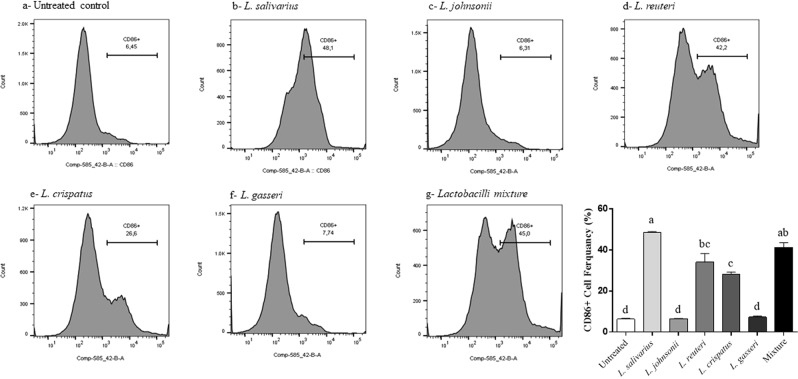
Expression of macrophage costimulatory molecule CD86 following treatment with heat-killed lactobacilli. MQ-NCSU cells were seeded, in triplicates, into 24-well plates and then treated with 100 MOI of heat-killed lactobacilli. After 24 hours of incubation, cells were harvested and stained with anti-CD86 followed by anti-mouse IgG2a secondary antibodies. Data analysis was performed using FlowJo software. Graphical data are presented as mean ± standard error of the mean (SEM). Bars (within a time point) which are marked by the same letter did not differ significantly (Duncan’s multiple range tests, P > 0.05).

In this study, we explored the role of lactobacilli in enhancing the expression of costimulatory molecules that play a critical role in T cell activation by measuring the expression of CD40 CD80, CD86, and MHC-II following treatment with heat-killed lactobacilli. The results showed that treatment of macrophages with *L. salivarius, L. reuteri, L. crispatus*, and the mixture of the five species significantly increased the expression of CD40 (Fig. 9), CD80 (Fig. 10), and CD86 (Fig. 11), whereas no significant changes were observed following treatment with *L. johnsonii* or *L. gasseri*, compared to the untreated controls. None of the treatments induced significant alterations in the surface expression of MHC-II molecules (data not shown).

## Discussion

Over the last few decades, probiotics have emerged as a promising alternative to antibiotics. In poultry, lactic acid bacteria (*Lactobacillus* and *Bifidobacterium* species) are the most commonly used probiotic organisms for prevention or control of many enteric diseases^4,5^. In addition to their roles in modulation of growth performance, probiotics have been shown to possess antimicrobial activity against various foodborne bacterial pathogens, such as *E. coli, Salmonella* spp., and *C. jejuni*^10,23,24^. Probiotics exhibit these activities either directly, through the production of bactericidal molecules, including organic acids, hydrogen peroxide, and bacteriocins, or indirectly, through competitive exclusion and/or the induction of innate mucosal immunity^25–27^.

Although there have been many studies examining the protective effects of lactobacilli against *C. jejuni*, the mechanisms underlying their protective activity and their interactions with the chicken immune system are not yet completely understood. A better understanding of these mechanisms may help reveal potential probiotic properties of lactobacilli and could also help identify the most effective probiotic strain(s), potentially leading to their use as feed additives to prevent *Campylobacter* colonization in broiler chickens. Therefore, the present study was undertaken to examine the probiotic potential of five *Lactobacillus* spp. for growth inhibition and virulence attenuation of *C. jejuni* and to evaluate their immunomodulatory properties.

The ability of lactobacilli to inhibit the growth of *C. jejuni* is thought to occur through the secretion of organic acids, such as lactic and acetic acids, which, in turn, alter the local pH to a level that makes the environment unsuitable for *C. jejuni* growth and/or causes disruption of the integrity of the *C. jejuni* membrane^28,29^. It has been shown previously that the cell-free supernatant of *L. fermentum* can inhibit the growth of *C. jejuni in vitro*; however, no such effect was found when the pH was increased to 6.3^30^. Strikingly, in the present study, both acidic and neutralized lactobacilli supernatants showed comparable levels of inhibition, indicating that the inhibitory capability of lactobacilli is not strongly pH-dependent, rather other antimicrobial substances may contribute to growth inhibition. Live lactobacilli, however, varied in their antagonistic activities against *C. jejuni* growth. It is noteworthy that *L. salivarius* displayed significant levels of bactericidal activity against *C. jejuni*, comparable to that of the mixture of the five *Lactobacillus* spp., indicating the differential abilities of *Lactobacillus* spp. to antagonize *C. jejuni*.

In addition to their bactericidal activity, lactobacilli may be able to attenuate *C. jejuni* virulence. Indeed, *C. jejuni* expresses several virulence factors, including those related to motility such as *flaA, flaB*^31,32^, *flhA*, and *flhB*^33^, adhesion such as *cadF*^34,35^, invasion such as *ciaB*^36^ and *iamA*^37^ cytotoxin such as *cdtA*^38^ and autoinducer production *luxS*^39^. Down-regulation of expression of virulence-associated genes in *C. jejuni* has been shown to attenuate its survival, colonization and invasion ability into chicken and human intestinal epithelial cells^40,41^. In the present study, all *Lactobacillus* spp., except *L. reuteri*, downregulated motility-associated genes. Since motility is required for *C. jejuni* to escape from stressful changes in the environment and to colonize the mucus layer lining the intestinal epithelium^35,42^, the observed reduction in the expression of motility-related genes and subsequent impairment of flagellar motility apparatus may consequently result in longer exposure of *C. jejuni* to antimicrobial substances produced by lactobacilli, thus suppressing its growth.

Invasion of human enterocytes is regarded as an important virulence factor of *C. jejuni*. Activation of invasion-related genes is required for translocation of *C. jejuni* across human enterocytes^43^. In view of this, the observed reduction in the expression of invasion-related genes following exposure to lactobacilli may subsequently reduce *C. jejuni* invasion of human enterocytes. This observation was further confirmed by examining the ability of *C. jejuni* to invade human intestinal epithelial cells in the presence or absence of lactobacilli. Our findings are consistent with those reported by Wine and colleagues in that lactobacilli have the potential to attenuate the invasive ability of *C. jejuni* against human intestinal epithelial cells^44^.

The expression of virulence genes that are involved in the pathogenesis of *C. jejuni* is coordinated by a quorum sensing regulatory system^45,46^. Quorum sensing is the process of cellular communication that allows bacteria to regulate their gene expression in accordance with cell density^45,47^. This regulation is mediated by diffusible signaling molecules, known as autoinducers^45,47^. AI-2 is an extracellular signaling molecule produced by many bacteria, including *C. jejuni*. In *C. jejuni*, the biosynthesis of AI-2 is catalyzed by the *luxS* gene product (S-ribosylhomocysteinase) and reaches a threshold level during the mid-exponential growth phase^48^. Alterations in *luxS* gene expression can, therefore, alter the production of AI-2. In the present study, we observed a concomitant reduction in the expression of *luxS* gene and AI-2 levels following exposure to lactobacilli, which is indicative of a disruption of bacterial quorum sensing signals. In addition to these effects, previous studies have also shown that the *luxS* gene is involved in the regulation of other virulence factors, including the transcription of the flagellin and cytolethal-distending toxin genes, biofilm formation and adherence to and invasion of human intestinal epithelial cells^46,49^. Therefore, it is conceivable to speculate that the administration of probiotic lactobacilli to chickens may not only reduce *C. jejuni* colonization, but could also attenuate the ability of *C. jejuni* to survive, produce cytotoxins, and invade human intestinal epithelial cells, thereby reducing the incidence of human illness. To evaluate this hypothesis, further studies are needed to investigate the ability of *C. jejuni*, harvested from lactobacilli-treated chickens, to invade human intestinal epithelial cells *in vitro* or the intestine of a suitable animal model *in-vivo*.

The second aim of this study was to evaluate the immune-stimulatory effects of lactobacilli on chicken macrophages. Macrophages are known as the main effector cells of the innate immune system and represent the first cellular line of defense against invading pathogens^50^. Upon activation, macrophages can eliminate pathogens directly through phagocytosis and production of nitric oxide, or indirectly through antigen presentation and secretion of cytokines and other mediators^51^ which, in turn, initiate a cascade of events leading to activation of other cells of the immune system. In this context, mounting evidence indicates that intestinal macrophages can be activated by the commensal microbes and their metabolites^52^.

Due to the difficulty of obtaining sufficient numbers of macrophages from the chicken intestine, macrophage-like cells (MQ-NCSU) were used as an *in vitro* model to study the interplay between lactobacilli and the host immune system. Even though these cells may not be a true representation of chicken macrophages, several research groups, including our group, have shown that these cells possess similar features of the mononuclear phagocyte lineage and exhibit chicken macrophage biology and function^17,22,53–55^.

Given the prominent role of NO in the disruption of bacterial membrane integrity and eventual killing of bacteria, this study sought to determine the capacity of different *Lactobacillus* spp. to induce NO production in chicken macrophages. Consistent with a previous study showing that *L. acidophilus* and *L. salivarius*, but not *L. reuteri*, enhance production of NO by chicken macrophages^16^, we demonstrated that all *Lactobacillus* spp., except *L. reuteri*, enhanced the production of NO by macrophages. It should be noted that a higher concentration of *L. reuteri* significantly inhibited NO production by macrophages, which may explain the absence of synergy of the lactobacilli mixture, as compared with the untreated group.

A series of studies have indicated that probiotics can enhance macrophage phagocytosis *in vitro* and *in vivo*. Here, we extend previous findings demonstrating that *in vitro* treatment of macrophages with lactobacilli enhances the uptake of latex beads into macrophages^16,17,22^. Further, our results show that pre-treatment of chicken macrophages with lactobacilli enhances their phagocytic activity against *C. jejuni*, as demonstrated by the high number of internalized *C. jejuni* as compared to the untreated cells. In the context of enteric infection, a previous study in mice reported that oral administration of probiotic lactobacilli mediates *Candida albicans* phagocytosis and clearance by intestinal macrophages^56^. Another study in chickens postulated that intestinal macrophages activated by probiotics may contribute to protection against colonization by *Salmonella enterica* serovar Enteritidis^57^.

Collectively, the notable increase in NO levels coupled with the enhancement of macrophage phagocytosis suggests that oral administration of these lactobacilli spp. to chickens can potentially reduce intestinal colonization by *C. jejuni*, at least in part, through activation of tissue-resident gut macrophages.

The findings of the present study demonstrated that lactobacilli differentially altered cytokine expression profiles in macrophages. The simultaneous induction of both pro-and anti-inflammatory cytokines by lactobacilli is indicative of the unique immunomodulatory functions of these probiotic bacteria, as well as their potential to maintain immune system homeostasis in the host. We have previously shown that the induction of pro-inflammatory cytokines and chemokines in chicken ileum and cecal tonsil contributes to protection against colonization by *C. jejuni*^58,59^. Previous studies have also shown that protection against *Salmonella enterica* serovar Typhimurium correlates with probiotic-induced cytokine and antimicrobial peptide gene expression in cecal tonsils^15,25,60^. In view of these facts, it is tempting to speculate that the administration of probiotics to chickens can provide protection against colonization of *C. jejuni* through the induction of cytokine expression in intestinal innate immune system cells.

Recognition and elimination of pathogens requires an effective communication between the innate and adaptive immune systems^61^. For example, T cell activation is orchestrated by the cellular components of antigen presenting cells such as costimulatory molecules^62^. Thus, the enhanced cell-surface expression of CD40, CD80, and CD86 molecules in response to lactobacilli will, in turn, lead to a series of interactions between macrophages and T-cell surface molecules that ultimately culminate in adaptive immunity against invading pathogens.

Inconclusion, these findings demonstrated that lactobacilli exhibit differential antagonistic effects against *C. jejuni* and vary in their ability to stimulate innate responses in chicken macrophages. The failure of *L. reuteri* to attenuate *C. jejuni* virulence and to induce NO production in macrophages, indicates that not all lactobacilli possess desirable probiotic properties and may also explain the lack of synergistic effects of the lactobacilli mixture. Nevertheless, *L. reuteri* has demonstrated the ability to inhibit the growth of *C. jejuni* and to promote various sets of effector molecules present during innate responses. Despite these promising results, the *in vitro* models, used in this study, may not accurately mimic the intestinal environment. Thus, further research is warranted to ascertain the effects of these lactobacilli in a complex cellular interaction in an *in vivo* setting. Future studies will aim to assess the immune responses in gut lymphoid and epithelial cells following administration of probiotic lactobacilli and the potential use of these bacteria, either as single or multiple species, as feed additives to prevent *Campylobacter* colonization in broiler chickens.

## Materials and Methods

### Evaluation of antagonistic activity of lactobacilli against *C. jejuni*

The inhibitory activity of the cell-free supernatant of lactobacilli against *C. jejuni* was performed using radial diffusion assay as described previously by Arsi *et al*.^21^, with minor modifications. Briefly, 10^8^ CFUs of *C. jejuni* was mixed with 30 mL of MH agar and poured into a 100 mm round Petri dish. Approximately 3 mm diameter holes were punched in the agar and 20 μL of the naturally-acidic or neutralized cell-free supernatant of each *Lactobacillus* sp., or a mixture of all lactobacilli was added to the holes. After the supernatants were fully absorbed, plates were overlaid with 10 mL of MH agar and incubated at 41 °C under microaerophilic conditions. After an incubation period of 40–48 h, the diameters of the zones of inhibition were measured.

The killing assay was used to determine the bactericidal activity of both the live culture and cell-free supernatants of lactobacilli against *C. jejuni*. Briefly, equal volumes of the naturally-acidic or neutralized cell-free culture supernatant of each *Lactobacillus* sp., or the mixture of the supernatants and 10^7^ CFUs of *C. jejuni* in MH broth were co-incubated at 41 °C overnight under microaerophilic conditions of 10% CO_2_, 5% O_2_, and 85% N_2_. Similarly, equal volumes of 10^7^ CFUs of each *Lactobacillus* sp. or the mixture of all species in MRS and 10^7^ CFUs of *C. jejuni* in MH broth were co-incubated overnight under microaerophilic conditions. The *C. jejuni* culture alone was used as a positive control. Subsequently, 100 µL of each culture was serially-diluted and streaked onto MH agar. Plates were incubated at 41 °C under microaerophilic conditions and the CFUs of *C. jejuni* enumerated after 40–48 h of incubation.

### Effects of lactobacilli on the expression of virulence-related genes in *C. jejuni*

Equal volumes (500 µL) of 10^7^ CFUs of *C. jejuni* in MH broth and 10^8^ CFUs of each *Lactobacillus* sp., or 10^8^ CFUs of the mixture of all species, or MRS medium were incubated at 41 °C for 24 h under microaerophilic conditions.

Prior to RNA extraction, RNAprotect Bacteria Reagent (Qiagen 76506) was added to each culture (2:1) for stabilization of RNA in bacterial cultures and total RNA was subsequently extracted using TRIzol reagent (Invitrogen) according to the manufacturer’s protocol. cDNA synthesis was performed as previously described by Koolman *et al*.^35^.

Quantitative real time-PCR (qPCR) was used to measure the transcripts levels of six different housekeeping genes of *C. jejuni* (*gyrA, ilvC, rpoA, slyD, thiC* and *rrs* {16 s RNA}) as previously described by Ritz *et al*.^63^. Sequences of all primers are outlined in Table 2. A standard curve was created for each gene and the efficiencies of the primers were calculated using LightCycler® 480 Software (Roche Diagnostics GmbH, Mannheim, DE). SASqPCR was used to assess the PCR amplification efficiency and rank the stability of these genes as previously described^64^.

**Table 2:** Genes and primer sequences used for *C. jejuni* reference genes and virulence-related genes.

Target gene	Primer sequence	Reference
*gyrA*	F: GTTATTATAGGTCGTGCTTT R: CTATGAGGTGGGATGTTTGT	^63^
*ilvC*	F: GCATGCAGAACGCAAAAATA R: TGATCCAAGGCATCATAGCA	^63^
*rpoA*	F: CGAGCTTGCTTTGATGAGTG R: AGTTCCCACAGGAAAACCTA	^63^
*slyD*	F: TACGATGAAAATGCCGTTCA R: TTCGCCAAAAAGCTCCATAC	^63^
*rrs*	F: AAGGGCCATGATGACTTGAC R: AGCGCAACCCACGTATTTAG	^63^
*thiC*	F: TTATCTTTGGGCGATGCTTT R: CATCCCAAGCCCTTTGAGTA	^63^
*flaA*	F: GGATGGCGATAGCAGATAGTTT R: CTCATCCATAGCCTTATCAGCA	^67^
*flaB*	F: ACACCAACATCGGTGCATTA R: CATCCCTGAAGCATCATCTG	^67^
*flhA*	F: GGAGCGATTAAAGGCCCCAA R: AGTGGTGGCACTTGTCCAAA	^35^
*flhB*	F: CAGGTGCGGATGTGGTGATC R: CACTCCTTTGGCAACAACCCT	^35^
*cadF*	F: TTCTATGGTTTAGCAGGTGGAG R: TTACACCCGCGCCATAAT	^67^
*iamA*	F: GAAGATGCACTTGCTTTGCG R: ATACCGCCACTAAGTTCGCT	^35^
*ciaB*	F: AAAAGCTTGGCAAGAAGCTG R: ATGCCACCGCATGAGTATAA	^67^
*cdtA*	F: GGATTTGGCGATGCTAGAGTT R: CATTTGTGCGTGATTGCTTG	^67^
*luxS*	F: AAAATGCCAGCTCCTGCTGT R: GTGCGACAACCCATAGGTGA	^35^

Quantitative real time-PCR was used to measure the relative expression of genes responsible for motility such as *flaA, flaB, flhA*, and *flhB*, adhesion such as *cadF*, invasion such as *ciaB* and *iamA*, cytotoxin production such as *cdtA* and autoinducer production *luxS*. The PCR reactions and cycling conditions were previously described^35^ and sequences for all primers are outlined in Table 2. A standard curve was created for each gene and the efficiencies of the primers were calculated using LightCycler® 480 Software (Roche Diagnostics GmbH, Mannheim, DE). Expression levels of all genes were calculated relative to the selected reference gene, rrs (16S RNA ribosomal subunit), using the 2^−ΔΔCT^ method (LightCycler® 480 Software, Roche Diagnostics GmbH, Mannheim, DE).

### Effect of probiotics on *C. jejuni* autoinducer-2 production (quorum sensing)

The levels of extracellular AI-2 produced by *C. jejuni* were measured using the *Vibrio harveyi* bioluminescence assay as described by Carter *et al*.^65^. Briefly, the reporter strain *V. harveyi* BB170 and the positive control *V. harveyi* BB152 were grown overnight at 30 °C and diluted 1: 5,000 into autoinducer bioassay (AB) medium consisting of a mixture of two buffers (Buffer1: 0.3 M NaCl, 0.05 M MgSO4, 2% vitamin free casamino acids, pH 7.5; Buffer 2: 1 M KPH4, 0.1M L-arginine hydrochloride, 50% glycerol, pH 7.0). Equal volumes (500 µL) of 10^7^ CFUs of the mid-log culture of *C. jejuni* in MH broth and the mid-log culture of 10^7^ *Lactobacillus* sp., or the mixture of lactobacilli in MRS broth were incubated at 41 °C for 8, 24 and 48 h under microaerophilic conditions. In a 96-well black clear bottom plate, 90 µL of the diluted *V. harveyi* BB170 and 10 µL of the filtered cell-free culture supernatant of both the treated and untreated culture of *C. jejuni* were added to the wells. The supernatant from *V. harveyi* BB152 was used as a positive control and AB medium was used as a negative control. Subsequently, the plate was incubated at 30 °C for 24 h with continuous shaking using VictorTM Multilabel plate counter (Wallac, PerkinElmer Life Sciences Canada, Woodbridge, Ontario, Canada). Luminescence production was measured every hour and the maximal bioluminescence was observed at 13 h after incubation with the lactobacilli. Data were presented as the relative light unit (RLU) per unit of absorbance (cell density; OD 600 nm).

### Effects of lactobacilli on *C. jejuni* associated with Caco-2 cells

Caco-2 cells were seeded in 6-well plates at a density of 4 × 10^5^ cells/well in EMEM and incubated at 37 °C in a humidified 5% CO_2_ environment until 90% confluency was reached. Afterward, 10^7^ CFUs of FITC-labelled *C. jejuni* in EMEM were added alone or simultaneously with 10^7^ CFUs of each *Lactobacillus* sp. or a mixture of all species to the Caco-2 cells and incubated for 2, 5 and 8 h. Adherent cells were washed with DPBS (pH 7.4) and fixed with 4% paraformaldehyde. Cells were permeabilized with 0.1% NP40 in PBS and the nucleus was stained with 7-AAD dye. The cell-associated fluorescent *C. jejuni* was visualized by fluorescence microscopy.

### Effects of lactobacilli on nitric oxide (NO) production

MQ-NCSU cells were seeded in a 24-well plate at density 4 × 10^5^ cells/well in LM-HAHN medium and incubated for 3 h. Afterward, cells were stimulated with either a single or a mixture of heat-killed *Lactobacillus* spp. at a multiplicity of infection (MOI) of 10 or 100 in DMEM and incubated at 41 °C in a humidified 5% CO_2_ environment for 24 h. Supernatants were collected and NO production was measured by Griess assay (Promega, USA), according to the manufacturer’s protocol.

### Effects of lactobacilli on phagocytic activity of chicken macrophage-like cells

#### Using fluorescent latex beads

A phagocytosis assay kit (IgG FITC; Cayman Chemical Michigan, USA) was used to quantitatively assess the phagocytic activity of macrophages. Briefly, MQ-NCSU cells were seeded, in 6 replicates, into 96-well black clear bottom polystyrene plates at a density 1 × 10^5^ cells/well in LM-HAHN medium and incubated for 3 h at 41 °C in a humidified 5% CO_2_ environment. Cells were then stimulated with either a single or a mixture of heat-killed *Lactobacillus* spp. at a MOI of 10 of 100 in DMEM along with the Latex BeadsRabbit IgG-FITC complex (according to manufacturer’s instruction) and incubated at 41 °C in a humidified 5% CO_2_ environment. After a 2 h incubation, cells were centrifuged at 400 × *g* for 10 min and the supernatant was discarded. Afterward, 50 μL of trypan blue solution was added to the cells, followed by incubation for 1–2 min at room temperature. Cells were then centrifuged at 400 × *g* for 10 min and excess trypan blue was removed. Fluorescence intensity was read using a fluorescence plate reader (PerkinElmer multimode plate reader, USA) at an excitation of 485 nm and an emission of 535 nm.

#### Using FITC-labelled *C. jejuni*

MQ-NCSU cells were seeded into 6-well plates containing glass coverslips at density 8 × 10^5^ cells/well in LM-HAHN medium as described above. After treatment with 100 MOI of either a single or a mixture of heat-killed lactobacilli, cells were incubated at 41 °C in a humidified 5% CO_2_ environment for 2 h. Subsequently, cells were washed three times with DPBS prior to infection with 8 × 10^7^ CFUs of FITC-labeled *C. jejuni*/well in DMEM medium. Cells were incubated for an additional 2 h and then washed three times with PBS. Adherent cells were washed with DPBS (pH 7.4) and fixed with 4% paraformaldehyde. Cells were permeabilized with 0.1% NP40 in PBS and the nucleus was stained with 4′,6-diamidino-2-phenylindole (DAPI). The internalization of fluorescent *C. jejuni* was visualized by fluorescence microscopy.

### Effects of lactobacilli on cytokine gene expression

MQ-NCSU cells were seeded in 24-well plates as described above. Cells were harvested at 3, 6 and 18 h post-treatment and RNA was extracted, and reverse transcribed to cDNA as previously described^59^. Expression levels of all target genes were calculated relative to the housekeeping gene β-actin using the 2^−ΔΔCT^ method (LightCycler® 480 Software, Roche Diagnostics GmbH, Mannheim, DE). The primers used in this study are outlined in Table 3. The PCR reactions and cycling conditions have been previously described^66^.

**Table 3:** Genes and primer sequences used for cytokines gene expression.

Target gene	Primer sequence (5′-3′)	Annealing Temp (°C)	Reference
IFN-γ	F:ACACTGACAAGTCAAAGCCGCACA R:AGTCGTTCATCGGGAGCTTGGC	60	^26^
IL-1β	F:GTGAGGCTCAACATTGCGCTGTA R:TGTCCAGGCGGTAGAAGATGAAG	64	^68^
IL-12p40	F: CCAAGACCTGGAGCACACCGAAG R: CGATCCCTGGCCTGCACAGAGA	60	^68^
CXCLi2	F:CCAAGCACACCTCTCTTCCA R:GCAAGGTAGGACGCTGGTAA	64	^68^
IL-10	F:TTTGGCTGCCAGTCTGTGTC R:CTCATCCATCTTCTCGAACGTC	64	^58^
β-actin	F:CAACACAGTGCTGTCTGGTGGTA R:ATCGTACTCCTGCTTGCTGATCC	60	^68^

### Effects of lactobacilli on the expression of macrophage cell surface proteins

Flow cytometry was used to determine the expression of CD40, CD80, CD86, and major histocompatibility complex (MHC)-II molecules following treatment with heat-killed lactobacilli as previously described^22^. Briefly, MQ-NCSU cells were seeded, in triplicates, into 24-well plates followed by being treated with 100 MOI of either a single or a mixture of heat-killed lactobacilli. After a 24 h incubation, the cells were stained in two different panels due to the paucity of having multi-colors in our staining panel. First panel used anti-MHC-II antibodies (directly labelled with Fluorescein isothiocyanate, FITC) and anti-CD40 (indirectly stained with anti-mouse IgG1 labelled with phycoerythrin, PE secondary antibodies). The second panel used anti-CD80 and anti-CD86 that were indirectly stained with anti-mouse IgG1- PE and anti-mouse IgG2a- FITC secondary antibodies, respectively. All the antibodies were purchased from AbD Serotec, NC. Dead cells were stained with the viable dye, Live/Dead stain near IR (infrared), purchased from Invitrogen, CA. Briefly, cells were re-suspended in staining buffer (PBS with 1% BSA) with the antibodies added and cells were stained for 30 min on ice and then washed and fixed with 2% paraformaldehyde before data acquisition on BD Canto-II flow cytometer. The gating strategy included excluding doublet cells through forward and side scatters height and width followed by gating on live cells excluding dead cells. Data analysis was carried out using the FlowJo software (Tree Star, Ashland, OR).

### Statistical analysis

All analyses were carried out using SAS version 9.3 (SAS, Cary, NC). The effects of lactobacilli on the growth of *C. jejuni*, virulence-related gene expression, *C. jejuni* associated with Caco-2 cells, phagocytic activity of macrophages, cytokine gene expression, and the expression of macrophage surface proteins were analyzed using SAS Proc GLM (General Linear Model), followed by Duncan’s multiple range test. The effects of lactobacilli on AI-2 and NO production were analyzed using Kruskal-Wallis followed by Wilcoxon. ImageJ software (https://imagej.net/Downloads) was used to measure the fluorescence intensity of the phagocytosed *C. jejuni* and to count the number of *C. jejuni* associated with Caco-2 cells. Data are presented as mean ± standard error of the mean (SEM) using GraphPad Prism V5.0 (GraphPad software, San Diego, CA, USA). *P* < 0.05 was considered significant for all statistical tests.
